# Curcumin prevents the bile reflux‐induced NF‐κB‐related mRNA oncogenic phenotype, in human hypopharyngeal cells

**DOI:** 10.1111/jcmm.13701

**Published:** 2018-06-17

**Authors:** Dimitra P. Vageli, Sotirios G. Doukas, Todd Spock, Clarence T. Sasaki

**Affiliations:** ^1^ Department of Surgery The Yale Larynx Laboratory Yale School of Medicine New Haven CT USA

**Keywords:** bile, curcumin, gastroesophageal reflux, head and neck cancer, NF‐κB

## Abstract

The presence of bile is not an uncommon finding in acidic oesophageal and extra‐oesophageal refluxate, possibly affecting the hypopharyngeal mucosa and leading to neoplastic events. We recently demonstrated that acidic bile (pH ≤ 4.0) can induce NF‐κB activation and oncogenic mRNA phenotype in normal hypopharyngeal cells and generate premalignant changes in treated hypopharyngeal mucosa. We hypothesize that curcumin, a dietary inhibitor of NF‐κB, may effectively inhibit the acidic bile‐induced cancer‐related mRNA phenotype, in treated human hypopharyngeal primary cells (HHPC), supporting its potential preventive use in vivo. Luciferase assay, immunofluorescence, Western blot, qPCR and PCR microarray analysis were used to explore the effect of curcumin in HHPC exposed to bile (400 μmol/L) at acidic and neutral pH. Curcumin successfully inhibited the acidic bile‐induced NF‐κB signalling pathway (25% of analysed genes), and overexpression of NF‐κB transcriptional factors, c‐REL, RELA(p65), anti‐apoptotic bcl‐2, oncogenic TNF‐α, EGFR, STAT3, WNT5A, ΔNp63 and cancer‐related IL‐6. Curcumin effectively reduced bile‐induced bcl‐2 overexpression at both acidic and neutral pH. Our novel findings suggest that, similar to pharmacologic NF‐κB inhibitor, BAY 11‐7082, curcumin can suppress acidic bile‐induced oncogenic mRNA phenotype in hypopharyngeal cells, encouraging its future in vivo pre‐clinical and clinical explorations in prevention of bile reflux‐related pre‐neoplastic events mediated by NF‐κB.

## 1 INTRODUCTION

Hypopharyngeal cancer is considered one of the most aggressive forms of head and neck malignancies with poor prognosis.^1^, ^2^ There is evidence that bile‐containing duodenal contents are present in aspirates of patients with gastroesophageal reflux disease (GERD)^3^ and growing epidemiologic evidence supports their involvement in laryngopharyngeal neoplasia.^4^, ^5^, ^6^, ^7^ Covington et al purport that the prevalence of bile in GERD may be greater than is believed by previous studies.^8^ Moreover, 24‐hour ambulatory pH monitoring in the pharynx of patients with GERD demonstrates that a drop below pH 4.0 is not uncommon and is considered diagnostic of a reflux event,^9^, ^10^ suggesting that acid may contribute to gastroduodenal‐induced inflammatory and neoplastic events, while pepsin had little effect in generating an oncogenic response^11^ Our recent in vitro and in vivo models have demonstrated that bile at acidic pH (≤4.0) is capable of inducing activation of NF‐κB and its signalling pathway in normal human hypopharyngeal cells, accelerating the expression of genes with anti‐apoptotic or oncogenic function, previously linked to HNSCC.^12^, ^13^, ^14^

The application of a specific pharmacologic NF‐κB inhibitor, BAY 11‐7082, significantly prevents the transcriptional activation of the acidic bile‐induced cancer‐related mRNA phenotype, suggesting a possible role of NF‐κB as a key mediator of acidic bile‐stimuli and early molecular oncogenic events in treated human hypopharyngeal epithelial cells.^15^, ^16^, ^17^

The crucial role of NF‐κB, as link between inflammatory and neoplastic events in epithelial cells, has been mechanistically linked to head and neck and other cancers.^18^, ^19^ NF‐κB is a transcriptional factor consisting of five subunits, RELA(p65), RELB, NFkB1, NFkB2 and c‐REL, and their heterodimeric complexes. NF‐κB complexes retained by IκB (composed of α and β subunits) are inactive in cytoplasmic forms. NF‐κB activation includes phosphorylation of its inhibitor by IKK (inhibitor kappa B kinase) resulting in IκBα ubiquitization and degradation releasing phopspho‐NF‐κB to translocate to the nucleus where it transcriptionally activates its target genes.^18^ NF‐κB is the first signalling pathway responding to harmful cellular stimuli, with fairly rapid alterations in gene expression, associated with pro‐tumorigenic events in cancers related to chronic inflammation.^20^, ^21^ The most important mechanism in which NF‐κB takes part in tumorigenesis is induction of anti‐apoptotic gene expression.^22^

Several pharmacologic and dietary inhibitors of NF‐κB are considered promising therapeutic options with chemo‐preventing or chemo‐sensitizing properties in head and neck cancer.^23^, ^24^ Curcumin is a turmeric natural supplement with known anti‐oxidant, anti‐inflammatory and anti‐cancer properties, previously shown to have potential chemopreventive effects in head and neck malignancies,^25^ blocking NF‐κB activation and halting the proliferation of cancer cells.^26^ However, the precise molecular mechanism by which curcumin inhibits NF‐κB and its related oncogenic function is not well understood. There is evidence that curcumin prevents nuclear translocation of NF‐κB by blocking the IKK‐mediated phosphorylation and degradation of IκBα, suppressing the expression of a variety of NF‐κB regulated gene products involved in carcinogenesis.^25^ Marquardt et al, showed that the growth inhibitory effect of curcumin in cancer cell lines is directly associated with NF‐κB‐related down‐regulation of phosphorylated p65 (p‐p65), JNK, Cyclin D1 and STAT3.^27^ Further evidence validating the action of curcumin in its anti‐cancer action is supported by its effect in deregulating an abundance of gene sets involved in IKK inhibition.^27^ Furthermore, extensive genomic analysis has revealed that anti‐cancer properties of curcumin associated with its powerful effect on pro‐oncogenic genes can induce a pro‐apoptotic effect by suppressing NF‐κB‐mediated cell survival pathways.^20^, ^21^ Precisely, because of its pleiotropic properties, curcumin is suggested to be more effective than single pathway targeted anti‐cancer strategies.^28^ In this manuscript, we hypothesize that curcumin can successfully prevent the acidic bile‐induced NF‐κB‐related oncogenic mRNA phenotype in human hypopharyngeal primary cells (HHPC).

We have undertaken this line of inquiry to (i) explore possible mechanistic similarities or differences to pharmacologic NF‐κB inhibition (ii) support potential pre‐clinical explorations of curcumin in preventing the oncogenic bile reflux effect.

## 2 MATERIALS AND METHODS

### 2.1 Cell culture

We cultured HHPC from Celprogen Inc. CA, USA, as previously described by Vageli D et al.^15^ HHPC was selected for this study based upon prior investigations demonstrating that HHPC effectively responds to pharmacologic NF‐κB inhibitor BAY 11‐7082, under acidic bile exposures.^15^, ^17^

### 2.2 Bile‐treatment

Human hypopharyngeal primary cells (2nd passage) underwent repeated exposure for 10 minutes, two times per day, for 4 days to (i) acidic bile (pH 4.0) and (ii) neutral bile (pH 7.0), containing 400 μmol/L of conjugated bile salts consistent with concentrations identified in patients (glycocholic acid: taurocholic acid: glycochenodeoxycholic acid: taurochenodeoxycholic acid: glycodeoxycholic acid: taurodeoxycholic acid; at molar concentration 20:3:15:3:6:1) (Sigma, St. Louis, MO; Calbiochem, San Diego, CA; USA) as previously described,^29^, ^30^ in DMEM/F12 10% FBS (Gibco^®^, NY, USA), (iii) acidic full growth media (pH of 4.0), considered as positive control and (iv) neutral full growth media (pH 7.0), considered as negative control. The media were removed and replaced with serum‐free media until the next exposure cycle (Human Hypopharyngeal Normal Cell Culture Media Serum Free; Celprogen Inc. CA, USA).

### 2.3 Bile + curcumin treatment

Human hypopharyngeal primary cell underwent an additional procedure of combined repetitive exposure to bile with curcumin (≥94% curcuminoid content; ≥80% curcumin; Sigma‐Aldrich), for 10 minutes, two times per day, for 4 days. Experimental and control groups underwent an identical procedure of repetitive exposure to “bile‐treatment,” as described above, in combination with 50 and 100 μmol/L curcumin.^26^ We also included additional control groups of untreated cells, used as a negative control and groups of cells repetitively exposed to DMSO, at concentrations similar to those used for curcumin solubilisation, as a reference control for the NF‐κB inhibitor vehicle. Cells of the experimental and control groups that were treated without inhibitor did not include a vehicle control.

We performed “Bile” and a “Bile + curcumin” treatment procedures in parallel*,* and at the end of treatment, media were removed, and cells or cell extracts were analysed.

### 2.4 Immunofluorescence assay

We performed an immunofluorescence assay, in HHPC, as previously described,^12^, ^15^, ^17^ to explore the effect of curcumin (100 μmol/L) on the acidic bile‐induced nuclear translocation of NF‐κB (p65 S536), linked to NF‐κB activation mediated by IKKβ and/or IKKα,^31^ as well as phospho‐STAT3 (Tyr705), previously shown to be up‐regulated in premalignant murine hypopharyngeal mucosa exposed to acidic bile.^13^

### 2.5 Western blotting

We performed a Western blot analysis, as previously described (Supporting Information),^15^ to determine the nuclear phospho‐NF‐κB (p65 S356) (~65 kD), and cytoplasmic phospho‐inhibitor‐kappaB‐a (p‐IκB‐α S32/S36) (~40 kD) and bcl‐2 (~28 kD) (p‐IκB‐α), protein levels on treated HHPC, with and without curcumin.

### 2.6 Luciferase assay

We performed a luciferase assay, using Firefly Luciferase Assay system (Promega Corporation, Madison, WI, USA), Lipofectamine^®^ 2000 (Invitrogen^TM^), pGL4.32[luc2P/NF‐κB‐RE/Hygro] Vector and control vector (pGL4.27[luc2P/minP/Hygro]), in order to monitor the activity of NF‐κB in HHPC exposed to bile with and without curcumin, as described in Supporting Information Methods.

### 2.7 Quantitative real‐time PCR

Real‐time quantitative polymerase chain reaction (qPCR) analysis was performed to evaluate the transcriptional levels of RELA (p65), c‐Rel, bcl‐2, TNF‐α, EGFR, STAT3, WNT5A, ΔNp63 and IL‐6, in HHPC exposed to bile with and without curcumin, as previously described (Supporting Information)^12^, ^15^ (Table S1). (Data were obtained from three independent experiments.)

### 2.8 Statistical analysis

Statistical analysis was performed using GraphPad Prism 6 software and one‐way ANOVA (Kruskal‐Wallis and Dunn's multiple analysis test; *P*‐values < .05) as well as *t* test analysis (multiple comparisons by Holm‐Sidak) to reveal any evidence of statistically significant reductions in protein or mRNA expression levels in different experimental and control groups treated by curcumin. We performed a Pearson correlation with estimate the correlation coefficient between the expression levels of different groups (*P*‐values < .05). Specifically, we used the Pearson analysis to identify correlations between the curcumin‐induced transcriptional levels of the analysed NF‐κB transcriptional factor, RELA (p65) and NF‐κB‐related genes, of the four different groups of treated HHPC.

### 2.9 PCR array for NF‐κB signalling pathway

We performed PCR microarray analysis of the NF‐κB signalling pathway in acidic bile‐treated groups with and without curcumin, to identify its effect on the acidic bile‐induced gene expression profiling of the NF‐κB signalling pathway, as previously described^15^ (Supporting Information Methods).

### 2.10 Cell viability assay

We performed a cell viability assay, using Cell Titer‐Glo^®^ Luminescent Cell Viability Assay (Promega), in order to monitor the effect of curcumin (100 μmol/L) on HHPC treated with bile at pH 4.0 and pH 7.0, compared to corresponding controls, as presented in Supporting Information Methods. We determined the mean values of cell viability with curcumin vs the mean value of cells without curcumin exposure, for each experimental and control group. Statistically significant difference of cell viability was determined using paired‐test and *P* value < .05 (Graph Pad Prism 6.0).

## 3 RESULTS

### 3.1 Curcumin inhibits acidic bile‐induced NF‐κB activation and bcl‐2 overexpression in HHPC

**Figure 1:**
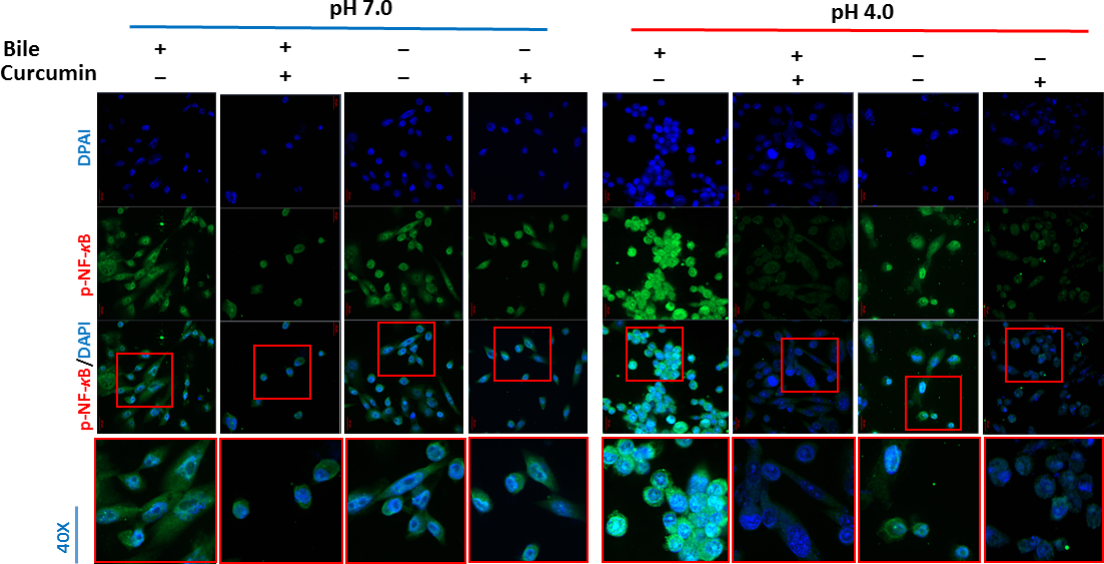
Curcumin inhibits the acidic bile‐induced nuclear translocation of NF‐κB (p65) phosphorylated at Ser536 in human hypopharyngeal primary cells (HHPC). Immunofluorescence staining for phospho‐NF‐κB (p‐p65 Ser536) reveals that application of curcumin inhibits the nuclear translocation of p‐p65 in acidic bile‐treated HHPC, demonstrating decreased p‐p65 nuclear levels (green: p‐p65 Ser536; blue: DAPI for nuclear staining)

**Figure 2:**
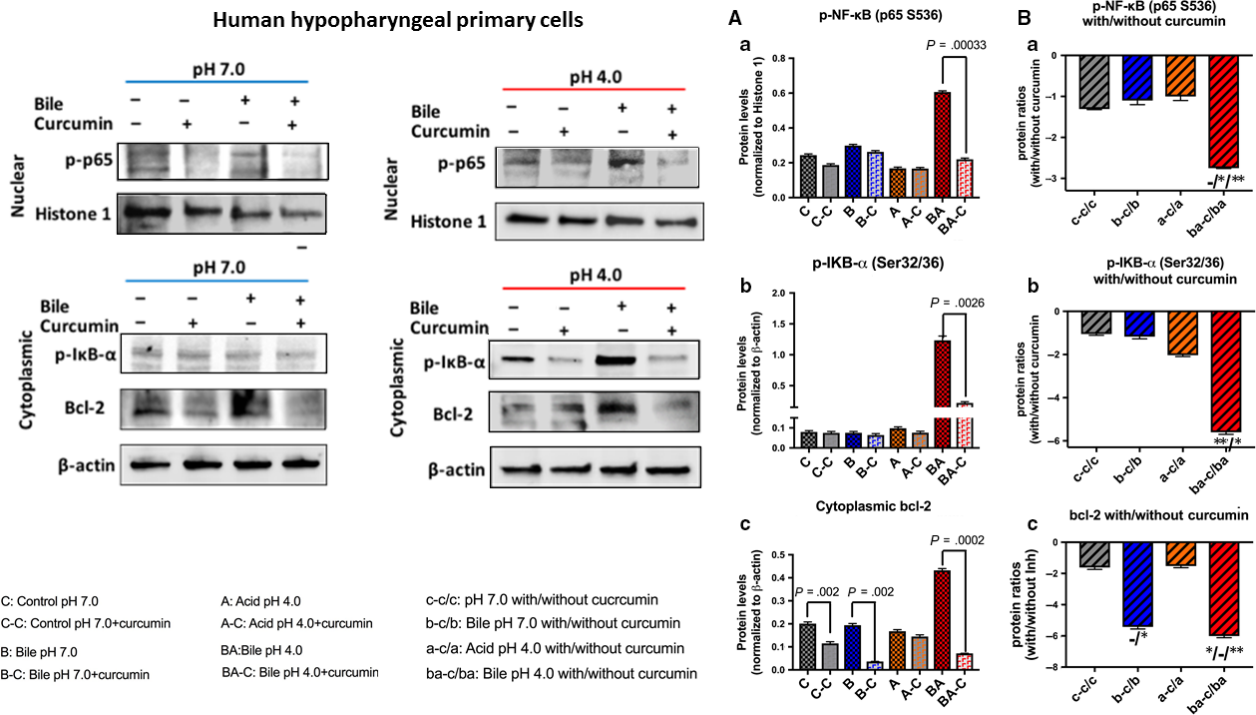
Curcumin inhibits the acidic bile‐induced NF‐κB activation and bcl‐2 overexpression in human hypopharyngeal primary cells (HHPC). Western blot analysis is performed in nuclear and cytoplasmic protein extracts of HHPC treated with and without curcumin, for p‐NF‐κB (p65 Ser536), p‐IκB‐α (Ser32/36) and bcl‐2. A, Normalized protein levels of (a) nuclear p‐NF‐κB(p65 Ser536), (b) cytoplasmic p‐IκB‐α (Ser32/36) and (c) cytoplasmic bcl‐2 (by *t* test; multiple comparisons by Holm‐Sidak; GraphPad Prism 6.0). B, Normalized protein ratios (with/without curcumin) of (a) nuclear p‐NF‐κB(p65 Ser536), (b) cytoplasmic p‐IκB‐α (Ser32/36) and (c) cytoplasmic bcl‐2, between different experimental and control HHPC‐treated groups (one‐way ANOVA; Kruskal‐Wallis, *P <* *.05; **P <* *.005; GraphPad Prism 6.0) (Nuclear p‐NF‐κB protein levels are normalized to histone 1; cytoplasmic p‐IκB‐α and bcl‐2 protein levels are normalized to β‐actin. Data are derived from three independent assays)

We observed that curcumin, a dietary inhibitor of NF‐κB, successfully inhibited acidic bile‐induced NF‐κB activation in treated HHPC. This observation was characterized by decreased p‐p65 nuclear staining, by immunofluorescence (IF) assay (Figure 1), as well as significantly decreased phospho‐NF‐κB nuclear levels, accompanied by reduced cytoplasmic p‐IκB‐α levels, by Western blot analysis, in cells exposed to bile at pH 4.0 (Figure 2A‐a,b) (*P *<* *.05; by paired *t* test; Graph Pad Prism 6.0).

Immunofluorescence assay also revealed that HHPC exposed to acid alone (pH 4.0) with curcumin demonstrated decreased p‐p65 nuclear staining, implying that curcumin blocks acid alone‐induced p‐p65 translocation to the nucleus (Figure 1). However, the effect of curcumin was less intense in HHPC exposed to acid alone, compared to acidic bile. This observation was supported by Western blot analysis, demonstrating that the effect of curcumin resulted in reduced nuclear p‐p65 and cytoplasmic p‐IκB‐α levels, in cells exposed to acid alone (pH 4.0), but without a statistically significant difference compared to those cells treated without curcumin (Figure 2A‐a,b).

We observed minimal changes in nuclear staining of p‐p65, as well as in cytoplasmic p‐IκB‐α levels, in HHPC exposed to neutral bile or neutral control with curcumin (pH 7.0), compared to neutral bile or neutral control without curcumin (Figures 1 and 2A‐a,b). Finally, we showed that cells exposed to DMSO, exhibited patterns of weak nuclear p‐p65 staining, implying that the solubilizing vehicle for curcumin had no effect on p‐p65 localization or expression (Figure S1).

We also found by Western blot analysis that curcumin suppressed cytoplasmic bcl‐2 accumulation in all treated groups. Specifically, we observed that curcumin prevented the acidic bile‐induced overexpression of bcl‐2 and also reduced cytoplasmic bcl‐2 accumulation in HHPC exposed to neutral bile (pH 7.0). This observation was characterized by a significant reduction of cytoplasmic bcl‐2 levels in HHPC exposed to bile at pH 4.0 or 7.0 with curcumin compared with those treated by bile at pH 4.0 or 7.0 without curcumin (Figure 2A‐c).

Further, we observed that HHPC exposed to acidic bile (pH 4.0) plus curcumin demonstrated the lowest relative expression ratios (with/without curcumin) of activated NF‐κB (Figure 2B‐a) and cytoplasmic p‐IκB‐α levels (Figure 2B‐b), with a significant difference compared with neutral bile (pH 7.0), acid alone (pH 4.0) or neutral control (pH 7.0) (*P *<* *.05; one‐way ANOVA, Kruskal‐Wallis, GraphPad 6.0). A significant reduction of cytoplasmic bcl‐2 levels was also observed in acidic bile‐treated HHPC with curcumin, compared to neutral control or acid alone (Figure 2B‐c). HHPC exposed to neutral bile plus curcumin also demonstrated lower relative expression ratios of bcl‐2 compared to neutral control (Figure 2B‐c).

Taken together, curcumin effectively prevented the acidic bile‐induced activation of NF‐κB, as previously shown by pharmacologic inhibitor of NF‐κB, BAY 11‐7082.^15^ We also observed that curcumin successfully prevented bile‐induced cytoplasmic accumulation of bcl‐2 in HHPC at either neutral or acidic pH.

### 3.2 Curcumin prevents acidic bile‐induced transcriptional activation of NF‐κB in HHPC

**Figure 3:**
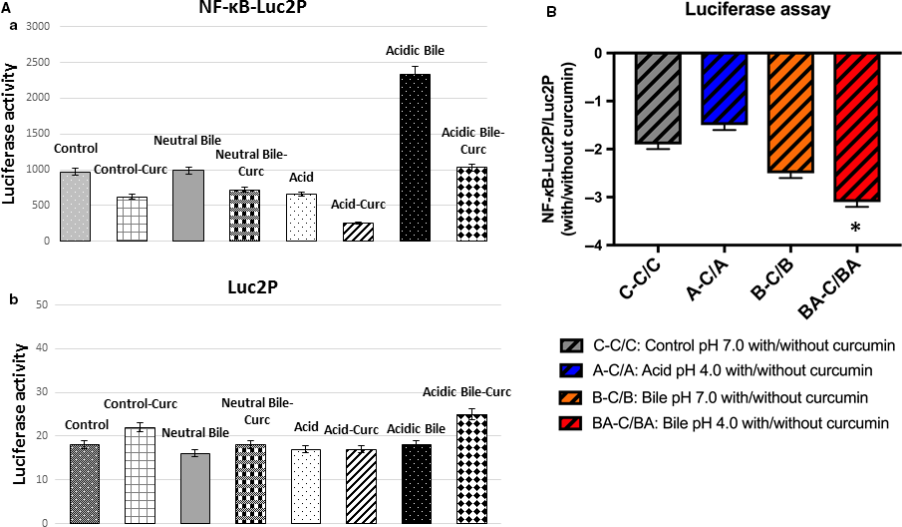
Luciferase assay demonstrates that curcumin suppresses the NF‐κB transcriptional activity in acidic bile‐treated human hypopharyngeal primary cells (HHPC). A, Columns of graphs represent luciferase activity (mean ± standard error of three independent experiments) in HHPC, transfected with NF‐κB luciferase responsive element (luc2P‐NF‐κB) and control luciferase reporter (luc2P). B, Columns of graphs represent NF‐κB relative transcriptional activity (Luc2P‐NF‐κB/Luc2P: NF‐κB luciferase responsive element/control luciferase reporter) in HHPC exposed to curcumin compared to those treated without curcumin. (*P <* *.05; one‐way ANOVA; Kruskal‐Wallis; GraphPad Prism 6.0)

Luciferase assay revealed that the acidic bile‐induced NF‐κB transcriptional activity was successfully inhibited by curcumin in HHPC (Figure 3). This was demonstrated by lower transcriptional activity NF‐κB in HHPC exposed to acidic bile with curcumin, compared to acidic bile without curcumin (Figure 3A) (NF‐κB‐luc2P: firefly luciferase reporter with NF‐κB responsive element, normalized to control luc2P: firefly luciferase reporter without NF‐κB responsive element). As shown by the negative ratios of relative NF‐κB activity in Figure 3B, HHPC, exposed to bile at pH 4.0 plus curcumin, exhibited the most intense inhibition of NF‐κB transcriptional activity, compared to other experimental media or controls.

### 3.3 Curcumin prevents acidic bile‐induced overexpression of NF‐κB—related genes with anti‐apoptotic or oncogenic function in treated HHPC

**Figure 4:**
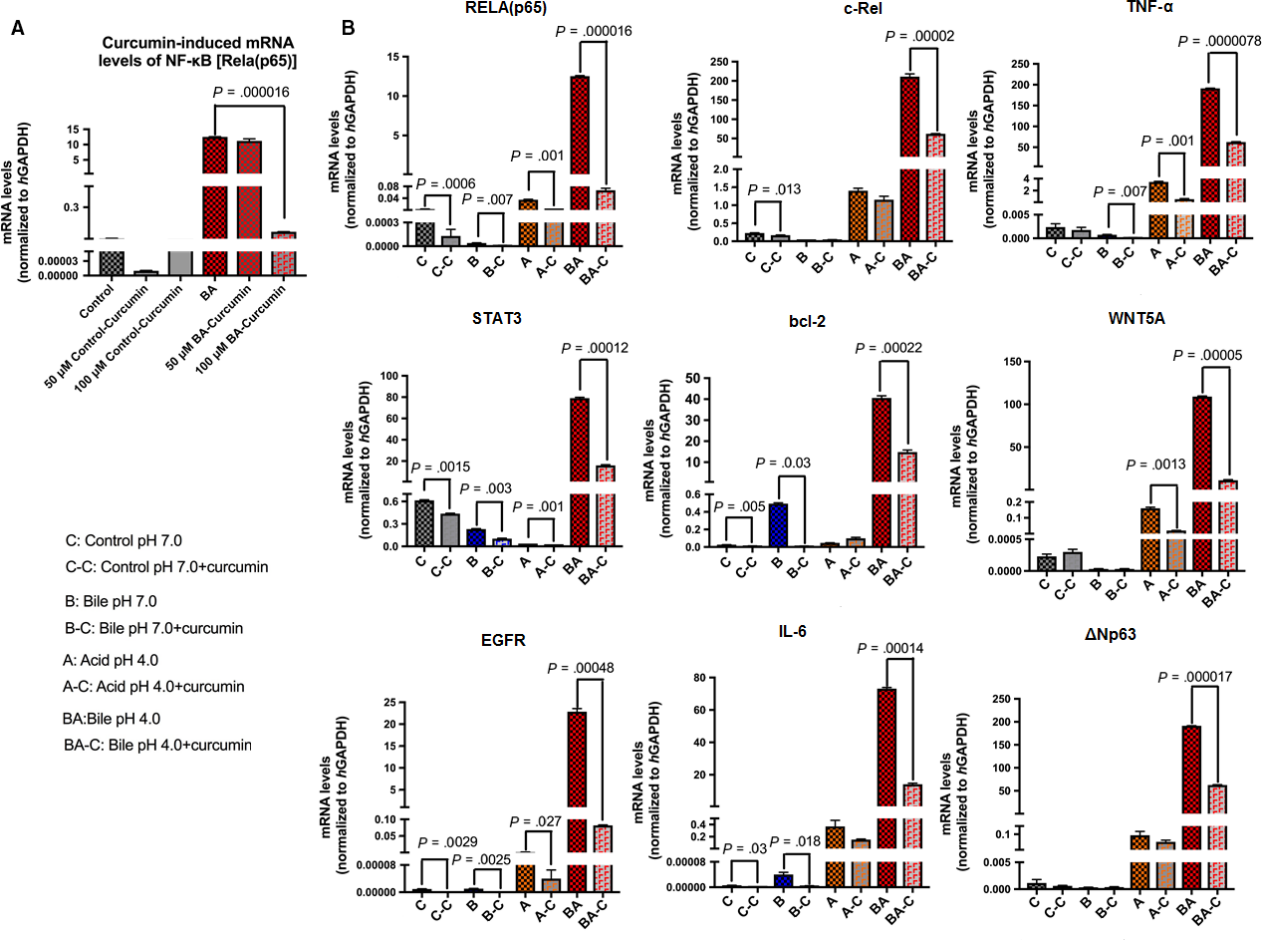
Curcumin prevents the acidic bile‐induced overexpression of genes with oncogenic function in treated human hypopharyngeal primary cells (HHPC). A, Graph represent transcriptional levels of NF‐κB transcriptional factor RELA (p65) in HHPC exposed to acidic bile with and without 50 and 100 μmol/L curcumin. B, Graphs represent transcriptional levels of each analysed gene, bcl‐2, EGFR, ΔNp63, c‐REL, RELA(p65), TNF‐α, STAT3, WNT5α and IL‐6 (relative to *h*GAPDH reference gene), in all experimental and control‐treated groups, with and without curcumin (100 μmol/L). (The data are derived from real‐time qPCR analysis. Data are derived from three independent experiments. Graphs, created by Graph Pad Prism 6 software, reveal transcriptional levels, normalized to *h*GAPDH, for the analysed genes between different experimental and control groups, in treated HHPC; by *t* test; multiple comparisons by Holm‐Sidak

Real‐time qPCR revealed that the acidic bile‐treated HHPC, without the dietary NF‐κB inhibitor, demonstrated the highest transcriptional levels of the analysed NF‐κB‐related genes with oncogenic function, RELA, c‐REL, bcl‐2, STAT3, EGFR, TNF‐α, WNT5A, ΔΝp63 and IL‐6 (Figure 4), in line with our prior studies.^13^, ^15^

Although curcumin at 50 μmol/L concentration had a minimal effect in reducing the mRNA levels of NF‐κB (RELA) (Figure 4A) and selected genes (Figure S2), curcumin at 100 μmol/L concentration, effectively prevented the acidic bile‐induced transcriptional activation of all the analysed genes, including NF‐κB (RELA), in treated HHPC (Figure 4A,B) (*P *<* *.05, by ANOVA). Specifically, we observed that curcumin (100 μmol/L) induced significantly lower transcriptional levels of each analysed gene, RELA(p65), c‐REL, bcl‐2, STAT3, EGFR, IL‐6, ΔΝp63, TNF‐α and WNT5A, in acidic bile‐treated HHPC (pH 4.0), compared to HHPC exposed to acidic bile without curcumin (Figure 4B) (*P *<* *.001; by *t* test; means ± SD; multiple comparisons by Holm‐Sidak).

**Figure 5:**
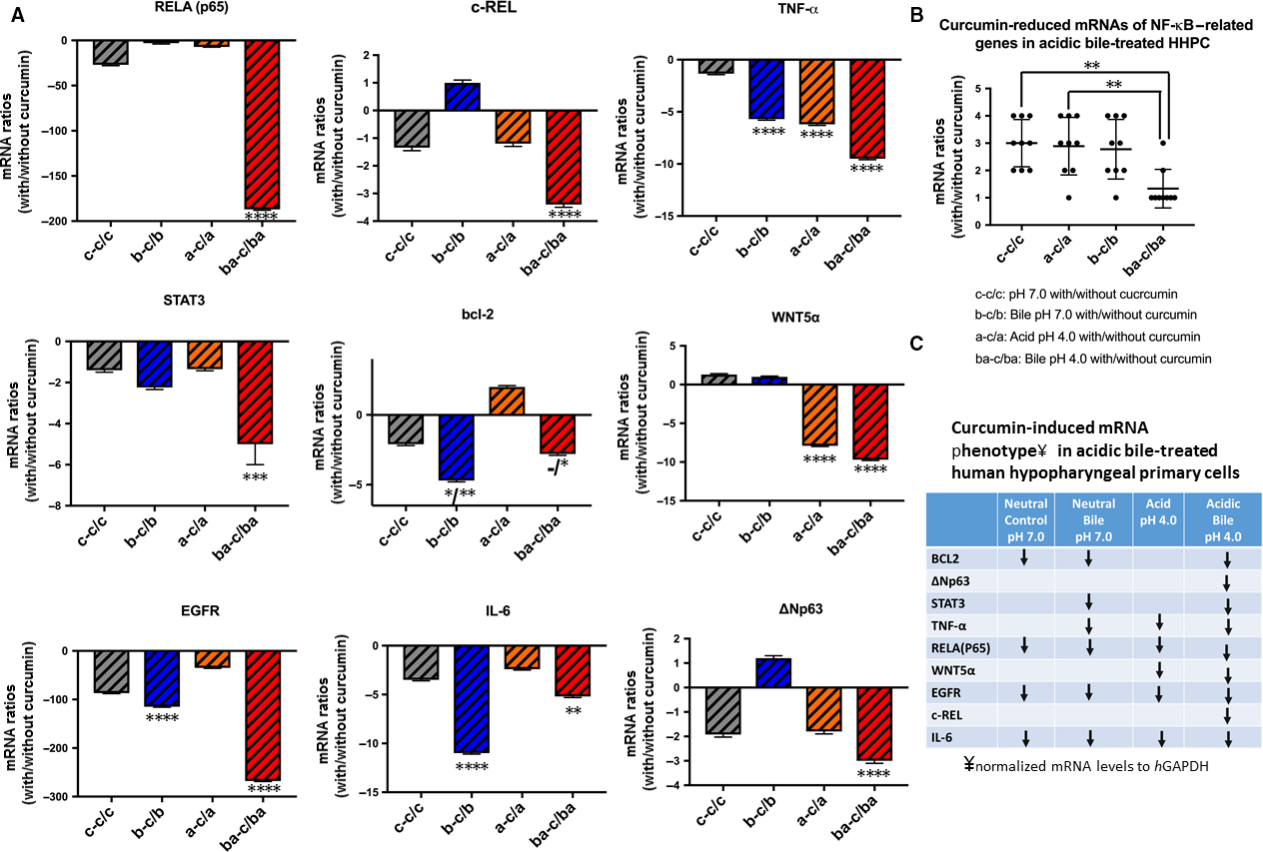
Relative mRNA expression ratios, with/without curcumin, of NF‐κB‐related genes with oncogenic function in acidic bile‐treated human hypopharyngeal primary cells (HHPC) and corresponding controls. A, Curcumin‐induced relative mRNA ratios (with/without 100 μmol/L of curcumin) of each analysed gene in different experimental and control groups of treated HHPC, by real‐time qPCR. B, Graph summarizes the relative mRNA ratios (with/without 100 μmol/L of curcumin) of all the analysed genes revealing that the HHPC exposed to acidic bile with curcumin produced the most significant reduction (with/without 100 μmol/L of curcumin), compared to other treated groups (neutral control/acid/neutral bile; *P <* *.05; **P <* *.005; ***P <* *.0005; ****P <* *.00005; one‐way ANOVA, Kruskal‐Wallis; Graph Pad Prism 6.0). C, Table describes curcumin‐induced mRNA phenotypes (≥2‐fold reduced mRNA ratios with/without curcumin) in treated HHPC. We demonstrate that curcumin in the acidic bile‐treated group produces a reduced mRNA phenotype for all analysed genes

Curcumin also reduced NF‐κB transcriptional factor RELA(p65), as well as oncogenic STAT3 and EGFR mRNAs in neutral bile, acid alone and neutral control‐treated cells (*P* < .01, by *t* test; means ± SD; multiple comparisons by Holm‐Sidak) (Figure 4A). However, HHPC exposed to acidic bile (pH 4.0) plus curcumin, exhibited the most intense mRNA reduction of RELA(p65), STAT3 and EGFR, as well as of c‐REL, TNF‐α, wnt5A and ΔNp63, compared to other experimental or control groups, as shown by the lowest negative ratios of relative mRNA expression (with/without curcumin) of these genes (Figures 5A and S2).

We also noted that the bile‐induced bcl‐2 overexpression was successfully suppressed by the applied dietary NF‐κB inhibitor (Figure 4B). This was demonstrated by the significant reduction of bcl‐2 mRNAs by curcumin in HHPC exposed to bile at acidic or neutral pH, compared to those treated without curcumin (Figure 5A).

We also noted a significant reduction of wnt5A by curcumin in HHPC exposed to acid alone or acidic bile, indicating that transcriptional activation of wnt5A was effectively suppressed by the dietary NF‐kB inhibitor under acidic conditions.

Taken together, curcumin successfully suppressed acidic bile‐induced overexpression of cancer‐related genes (Figure 4B), significantly reducing the mRNA profiling of all the analysed genes compared with controls (Figure 5B). Figure 5C shows that curcumin down‐regulates (≥2‐fold) the acidic bile‐induced mRNA phenotype including all of the analysed genes. However, a less intense effect of curcumin was observed in mRNA phenotypes of HHPC treated with neutral bile (pH 7.0) and controls (pH 7.0 and pH 4.0), with down‐regulation (≥2‐fold) of only a part of the analysed genes.

### 3.4 Curcumin inhibits, acidic bile‐induced nuclear translocation of p‐STAT3 in HHPC

**Figure 6:**
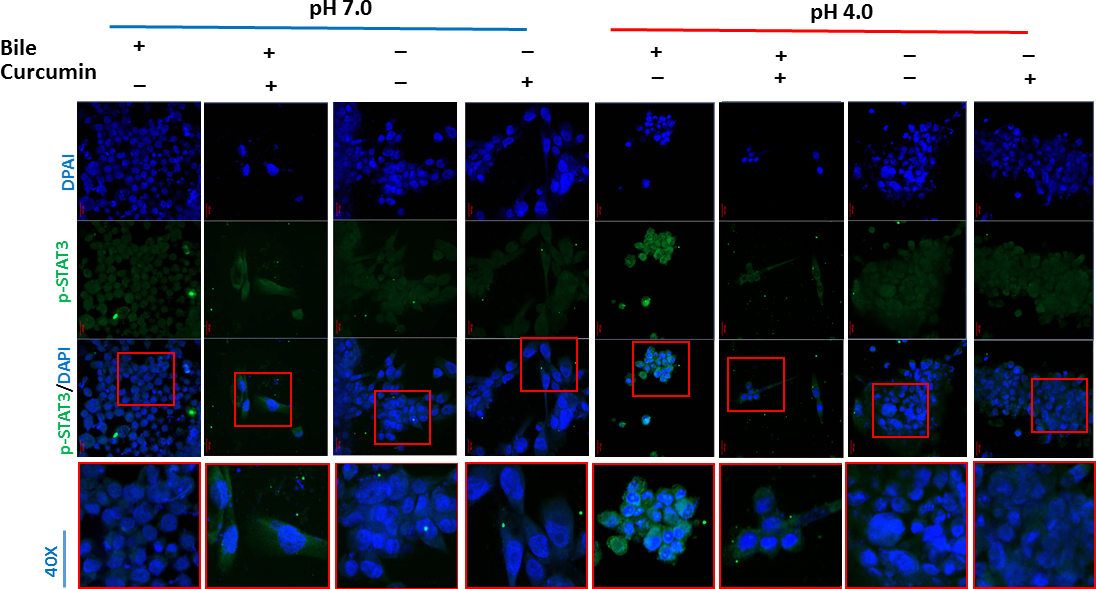
Curcumin inhibits acidic bile‐induced nuclear translocation of phospho‐STAT3 in human hypopharyngeal primary cells (HHPC). Immunofluorescence staining of phospho‐STAT3 (Tyr705) reveals that application of curcumin inhibits the acidic bile‐induced nuclear translocation of p‐STAT3 in HHPC, demonstrating decreased p‐STAT3 nuclear staining, compared to HHPC exposed to acidic bile without curcumin (green: pSTAT3 [Tyr705]; blue: DAPI for nuclear staining)

We further analysed the effect of curcumin in acidic bile‐induced activation of oncogenic STAT3, by performing IF assay for phospho‐STAT3 (Ty705) (Figure 6). We observed that curcumin inhibited p‐STAT3 nuclear translocation in acidic bile (pH 4.0) treated HHPC. On the contrary, only minor changes were noted in other experimental or control groups treated with curcumin. Specifically, we observed an intense nuclear and cytoplasmic staining for p‐STAT3 in acidic bile‐treated HHPC that was abolished in the presence of curcumin. Acid alone, neutral bile and neutral control‐treated cells demonstrated lower levels of nuclear p‐STAT3 compared with acidic bile‐treated cells. Therefore, curcumin had a minimal effect on p‐STAT3 levels in these groups, implying that curcumin specifically blocked acidic bile‐induced p‐STAT3 localization to the nucleus.

### 3.5 Correlations between curcumin‐induced transcriptional levels of NF‐κB and related genes in HHPC

We performed a *Pearson* analysis to determine correlations between the curcumin‐induced transcriptional activation of NF‐κB and mRNAs of NF‐κB‐related genes in treated HHPC (Figure S3). We observed a strong positive correlation between transcriptionally activated levels of NF‐κB and EGFR (*r *=* *.95073, *P *=* *.013) as well as NF‐κB and STAT3 (*r *=* *.91752, *P *=* *.0281) (Figure S3A).

*Pearson* analysis also revealed a significant positive correlation between curcumin‐induced mRNAs (i) RELA(p65) and EGFR (*r *=* *.93843, *P *=* *.0182) (ii) RELA(p65) and STAT3 genes (*r *=* *.95202, *P *=* *.0125) and (iii) EGFR and STAT3 (*r *=* *.98134, *P *=* *.0031). (iv) A positive but not significant linear correlation was also observed by *Pearson* between RELA(p65) and c‐REL (*r *=* *.83774) (Figure S3B).

### 3.6 Curcumin reduces acidic bile‐induced gene expression of the NF‐κB signalling pathway in HHPC

We performed a PCR array to explore the effect of curcumin in acidic bile‐induced gene expression profiling of NF‐κB signalling pathway. In the 84 analysed genes, we observed that acidic bile up‐regulated 66 of 81 informative analysed NF‐κB‐related genes (~83%) (>2‐fold change) (Table S3). Curcumin reduced the acidic bile‐induced transcriptional levels of 20 of 81 informative NF‐κB‐related genes (~25%) (>2‐fold change) (Table 1).

**Table 1:** Curcumin down‐regulates the acidic bile‐induced NF‐κB signalling in hypopharyngeal primary cell

Gene symbol	Fold regulation^a^	Gene symbol	Fold regulation	Gene symbol	Fold regulation
AGT	−1.7268	FOS	−1.7268	PSIP1	−1.7268
AKT1	−1.7268	HMOX1	−1.7268	REL	−1.7268
ATF1	−2.4065	ICAM1	−3.8161	RELA	−1.7268
BCL10	−1.7268	IFNA1	−1.7268	RELB	−1.7268
BCL2A1	−1.7268	IFNG	−1.7268	RHOA	1.6743
BCL2L1	1.0237	IKBKB	−1.7268	RIPK1	−1.7268
BCL3	−3.2942	IKBKE	−1.1376	STAT1	206.643
BIRC2	−3.3209	IKBKG	−1.7268	TBK1	−1.7268
BIRC3	−1.7268	IL10	−1.7268	TICAM1	−5.3155
CARD11	−65.866	IL1A	−1.7268	TICAM2	−1.7268
CASP1	−3.2932	IL1B	−1.7268	TIMP1	−4.2569
CASP8	−1.7268	IL1R1	−1.7268	TLR1	4.3961
CCL2	−1.7268	CXCL8	−1.7268	TLR2	−1.7201
CCL5	−4.2205	IRAK1	1.4956	TLR3	−1.7268
CD27	−1.4131	IRAK2	−1.7268	TLR4	−1.449
CD40	−1.7268	IRF1	−1.7268	TLR6	−1.134
CFLAR	−1.7268	JUN	−1.7268	TLR9	−1.7268
CHUK	−4.0318	LTA	−3.9297	TNF	−1.7268
CSF1	−1.7268	LTBR	−1.1619	TNFAIP3	−1.7268
CSF2	−7.1026	MALT1	−1.9853	TNFRSF10A	−1.7268
CSF3	−3.1932	MAP3K1	−1.7268	TNFRSF10B	1.2988
EGFR	−1.7268	MYD88	−1.7268	TNFRSF1A	−3.27
EGR1	−2.0815	NFKB1	−1.8091	TNFSF10	−5.451
ELK1	−1.7268	NFKB2	−23.798	TNFSF14	−1.7268
F2R	−3.4833	NFKBIA	−1.7268	TRADD	−1.7268
FADD	−1.7268	NFKBIE	−1.7268	TRAF2	−1.7268
FASLG	−1.7268	NOD1	−4.0366	TRAF3	−6.0591

a Up‐ and down‐regulation in acidic bile with curcumin (group 2), comparing to acidic bile without curcumin treated normal human hypopharyngeal cells (group 1).

Blue coloured values: down‐regulation; red coloured values: up‐regulation.
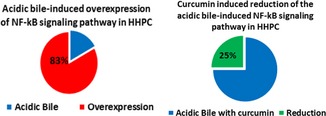

The effect of curcumin on the NF‐κB pathway and its genes is represented in Table S3. The most intense effect of curcumin included the down‐regulation of NF‐kB transcription factors, NFkB2 (>23‐fold), as well as members of TNF‐receptors, such as TNFRSF1A (>3‐fold), TNFSF10 (>5‐fold). Curcumin also reduced NF‐κB downstream signalling, preventing the expression of positive regulators of the NF‐κB pathway, such as BIRC2 (>3‐fold), CARD11 (>65‐fold), LTA (>3‐fold), TICAM1 (>5‐fold) and TRAF3 (>6‐fold). We found a high level of reduction in CARD11 (>65‐fold), activator of NF‐κB. The effect of curcumin also reduced the expression of Inhibitor‐kappaB kinase, CHUK (IKKa) (>4‐fold), as well as of bcl‐3 (>3‐fold), which is a co‐activator of NF‐κB, preventing the cytoplasmic release of NF‐κB.

NF‐κB inhibition resulted in reduction of NF‐κB responsive genes in acidic bile‐treated cells. Specifically, we observed a reduction in the expression of, ATF1 (>2‐fold) and EGR1 (>2‐fold) transcriptional factors and TIMP1 (>4‐fold), an anti‐apoptotic factor that can also promote cell proliferation.

### 3.7 Curcumin reduces cell viability in bile‐treated normal HHPC

We performed a cell viability assay, to determine the effect of curcumin (100 μmol/L) on cells exposed to acidic and neutral bile (pH 4.0 and 7.0), relative to controls (pH 4.0 and 7.0). Curcumin exhibited a negative effect on cell viability of all treated HHPC (Figure S4). It reduced the acidic bile‐induced cell survival (Figure S4A‐a) and also reduced the percentages of viable cells in neutral bile‐treated HHPC (Figure S4A‐b) (*P *<* *.05, multiple *t* test) but preserving a sufficient percentage of normal human hypopharyngeal cells. Curcumin also exhibited a significant negative effect on cell viability with neutral control (pH 7.0) but found to be less intense than those in neutral bile group (Figure S4A‐a). Curcumin also exhibited a weak negative effect on cell viability with acid alone (pH 4.0) (Figure S4A‐b). DMSO had an insignificant negative effect on cell viability, indicated by similar percentages of viable cells compared with controls (Figure S4B).

## 4 DISCUSSION

There is growing evidence that bile‐containing enterogastric reflux may be much more common than previously appreciated.^8^ It has been shown that during extra‐oesophageal reflux episodes, duodenogastric fluid reaches the epithelium of the upper aerodigestive tract and contributes to the development of inflammatory and neoplastic events.^4^, ^5^, ^6^ However, the role of bile‐containing extra‐oesophageal refluxate in hypopharyngeal cancer is not yet fully understood, and the underlying mechanism of its carcinogenic effect remains unclear. There is evidence that in head and neck cancer, the NF‐κB transcriptional factor is constitutively activated and linked to activation of known oncogenic pathways, such EGFR/Ras/RAF/MAPK, Akt/PI3K/mTOR, ΙΚΚ/NF‐κB, STAT3 and wnt/β‐catenin.^32^, ^33^, ^34^, ^35^, ^36^, ^37^, ^38^, ^39^, ^40^, ^41^

We recently demonstrated, using in vitro and in vivo models, that application of a specific pharmacologic inhibitor, BAY 11‐7082 in acidic bile‐treated hypopharyngeal epithelial cells, could block NF‐κB activation and prevent the acidic bile‐induced overexpression of cancer‐related mRNA phenotype, including STAT3, EGFR, bcl‐2, TNF‐α, ΔNp63, wnt5A and IL‐6.^15^, ^16^ Previous studies demonstrated that curcumin, a known dietary inhibitor of NF‐κB with anti‐inflammatory and anti‐cancer effect, could be used in chemoprevention of head and neck cancer.^24^, ^25^, ^26^ Here, we explored the effect of curcumin, in preventing acidic bile‐induced NF‐κB activation and overexpression of cancer‐related mRNA phenotype in treated HHPC. HHPC was used for this study based upon our prior investigation demonstrating that HHPC effectively responded to pharmacologic NF‐κB inhibitor BAY 11‐7082 under acidic bile exposures.^15^, ^16^

Our novel findings showed that, similar to the pharmacologic NF‐κB inhibitor BAY 11‐7082, the dietary NF‐κB inhibitor curcumin, successfully prevented the acidic bile‐induced overexpression of RELA(p65), c‐REL, bcl‐2, EGFR, STAT3, TNF‐α, ΔNp63, wnt5A and IL‐6. We showed that a higher concentration of curcumin, at 100 μmol/L, was required to effectively reduce the acidic bile‐related mRNA phenotype, while lower concentrations (50 μmol/L) had a minimal effect, supporting the observation that the inhibitory effect of curcumin was concentration dependent.^26^

Our data demonstrated that the effect of curcumin was significantly more intense in preventing NF‐κB activation and related genes with oncogenic function, in the acidic bile‐treated group (pH 4.0) compared to others. However, a fraction of the analysed genes was also affected by curcumin in neutral bile, acid alone and control‐treated groups. This observation suggested that although curcumin seemed to have a non‐selective effect, it was particularly successful in blocking acidic bile‐related early molecular events, previously observed in acidic bile‐induced premalignant hypopharyngeal mucosa.^13^

Our novel findings confirmed that curcumin reduced the acidic bile‐induced NF‐κB signalling pathway. As expected, curcumin down‐regulated a lower percentage of NF‐κB signalling analysed genes, compared to BAY 11‐7082 (25% vs 85%), a specific pharmacologic NF‐κB inhibitor.^15^ It is our view, however, that the less intense effect of curcumin compared to BAY 11‐7082 might confer a clinical advantage, preventing generalized suppression of NF‐κB signalling shown essential to basic metabolic function in healthy mucosa and thereby reducing global toxicity.^42^ Furthermore, curcumin was found capable of selectively inhibiting bile‐induced bcl‐2 overexpression in all treated groups, and particularly in HHPC exposed to bile at both acidic (pH 4.0) and neutral (pH 7.0) pH. Therefore, curcumin might be capable of providing an advantage over other NF‐κB inhibitors, preventing the bile‐related anti‐apoptotic effect, independently of pH status.^15^ These findings are supported by the reduced cell viability of bile‐treated HHPC in the presence of curcumin, at either acidic or neutral pH, further suggesting either stimulation of apoptotic pathways or suppression of NF‐κB‐mediated cell survival responses.^20^

Again our data showed that application of curcumin resulted in a successful suppression of acidic bile induced overexpression of NF‐κB and other crucial oncogenic factors, such as EGFR and STAT3, in treated HHPC, in line with previous studies demonstrating that curcumin inhibited NF‐κB and oncogenic EGFR and STAT3 in head and neck cancer.^23^ Specifically, NF‐κB/STAT3 crosstalk had been shown fundamental in inflammation‐associated carcinogenesis in head and neck cancer.^43^ Our findings supported this relationship demonstrating that NF‐κB inhibition could effectively decrease the expression of genes with cell proliferation or anti‐apoptotic function in HNSCC.^18^, ^23^, ^26^ Moreover, the successful inhibition of acidic bile‐induced nuclear translocation and transcriptional activation of both IL‐6 and STAT3 by curcumin in treated HHPC supported the view that the acidic bile‐induced activation of IL‐6/STAT3 is NF‐κB dependent.^43^ Finally, our data demonstrated that curcumin had a pronounced effect in suppressing the acidic bile induced overexpression of Wnt5A, a factor related to cancer‐associated inflammation and epithelial‐mesenchymal transition.^44^

Our recently published data suggest that NF‐κB inhibition is also capable of preventing acidic bile‐induced alterations of cancer‐related miRNA markers with potential regulatory role in acidic bile‐related oncogenic mRNA phenotype, such as miR‐21, miR‐155, miR‐192, miR‐34a, miR‐375 and miR‐451a.^17^ Data from our current study showing that curcumin was an effective inhibitor of acidic bile‐induced activation of NF‐κB, encourages its use, alone or in combination with BAY 11‐7082, in further explorations of the regulatory role of miRNA markers in acidic bile‐related mRNA oncogenic phenotype.

The exact composition of refluxate present in the upper aerodigestive tract of patients with extra‐oesophageal reflux disease has not yet been fully characterized. In the current in vitro model, we used a mixture of conjugated bile salts previously identified in oesophageal refluxate.^29^, ^30^ This specific composition was found to induce early pre‐neoplastic lesions in treated murine hypopharyngeal mucosa with an acceleration of NF‐κB‐activated gene expression profiles.^13^ Other studies suggest that extra‐oesophageal refluxate may contain unconjugated bile acids due to the de‐conjugating effect of gastric microflora,^45^ encouraging further investigation using physiologic concentrations of unconjugated bile acids.

Although, our novel in vitro data support the chemopreventive potential of curcumin on HHPC exposed to acidic bile, as we transition to in vivo models of exploration, we recognize the limitations of DMSO as a solubilizing vehicle. We will explore the use of nanotechnology‐driven presentations, to enhance the bioavailability of curcumin to targeted tissues,^28^, ^46^ increasing the effectiveness of orally administered curcumin in preventing bile reflux‐induced early pre‐neoplastic events. Additionally, it has been demonstrated that hydrophobic primary and secondary bile salts, such as sodium cholate and sodium deoxycholate, even at low concentrations, can facilitate the solubility and stability of curcumin and its delivery into the cells.^46^ These considerations suggest the potential in vivo use of curcumin in future explorations.

## 5 CONCLUSION

Our in vitro model demonstrates that bile‐related activation of NF‐κB, and its transcriptionally activated oncogenic factors can be inhibited by the use of turmeric supplements, such as curcumin, in exposed normal human hypopharyngeal cells, similar to the effect of specific pharmacologic inhibitor BAY 11‐7082^15^ again highlighting the importance of NF‐κB in generating the oncogenic phenotype. Our novel findings further suggest that selective dietary supplements with anti‐inflammatory and anti‐apoptotic properties without total NF‐κB ablation, may be useful for the prevention of extra‐oesophageal reflux‐related hypopharyngeal neoplasia, encouraging further investigation of their use in the prevention of acidic bile‐induced pre‐neoplastic events on topically exposed hypopharyngeal mucosa. Future in vitro and in vivo explorations using combinations of pharmacologic NF‐κB inhibitors and dietary inhibitors such as curcumin may reveal their additive or superadditive effect in inhibiting the NF‐κB‐related pathway or other molecular pathways effectively suppressing acidic bile‐induced oncogenic events and in so doing provide further means of mechanistically characterizing the NF‐κB effect in pro‐oncogenesis.

## CONFLICTS OF INTEREST

The authors whose names are listed in this article certify that they have no affiliations with or involvement in any organization or entity with any financial interest, or non‐financial interest in the subject matter or materials discussed in this manuscript.
