# Supplementary material for: Curcumin prevents the bile reflux‐induced NF‐κB‐related mRNA oncogenic phenotype, in human hypopharyngeal cells
Source: J Cell Mol Med. 2018 Jun 17;22(9):4209–20. doi: 10.1111/jcmm.13701 (PMC6111812; doi:10.1111/jcmm.13701)
Supplement: Supplementary file 1 [file JCMM-22-4209-s001.docx]

**Supplementary Information**

**Title: Curcumin prevents the bile reflux-induced NF-***κ***B-related mRNA oncogenic phenotype, in human hypopharyngeal cells**

Dimitra P. Vageli^1^, Sotirios G. Doukas^1^, Todd Spock^1^ and Clarence T. Sasaki^1^*

^1^The Yale Larynx Laboratory, Department of Surgery, Yale School of Medicine, New Haven, CT, USA

*corresponding author

**Supplementary Methods**

**Western blotting**

At the end of treatment 10 to 30 µg of HHPC cytoplasmic and nuclear protein extracts were heated at 70°C for 10 minutes in sodium dodecyl sulfate polyacrylamide gel electrophoresis Laemmli sample buffer (Bio-Rad, Hercules, CA), and were separated using 4-20% Mini-PROTEAN TGX Tris/Glycine pre-cast gels, at 150V for 1 hour, while Precision Plus Prestained Protein Standards (Dual Color or Kaleidoscope, *Bio-RAD*) were used providing a 10-band ladder (250-10 kD). Proteins were transferred onto a 0.45 mm nitrocellulose membrane, using Trans Blot Turbo transfer system (Bio-Rad), blocked in 5% BSA, for 1 hour, and were incubated with primary antibodies, of primary anti-phospho-NF-*κ*B (p65 Ser536; rabbit polyclonal anti-phospho-p65 Ser536, AbD Serotec, BIO-RAD, CA, USA), phospho-IκB-α Ser32/36 (5A5; Cell Signaling, EMD Millipore, Billerica, MA), and bcl-2 (C-2; Santa Cruz Biotechnology), which were diluted in 5% BSA, overnight at 4 °C. Membranes were incubated for 1:30 hours with goat anti-rabbit or anti-mouse horseradish peroxidase conjugated secondary antibodies (EMD Millipore) at 1:5000 and chemiluminescence was determined using an enhanced chemiluminescence detection system (Clarity Western ECL Substrate, Bio-Rad). Membranes also were stripped using Restore stripping buffer (Pierce) and were reported with β-actin (C4; Santa Cruz Biotechnology) for cytoplasmic extracts and Histone 1 (AE-4; Santa Cruz Biotechnology) for nuclear extracts normalization. Protein levels were quantified by Gel imaging system (*BIO-RAD*). in each nuclear or cytoplasmic cellular compartment, and expression levels were estimated by Image Lab 5.2 analysis software (*BIO-RAD*).

**Luciferase assay**

We used Firefly Luciferase Assay system (Promega Corporation, Madison, WI, USA), Lipofectamine® 2000 (Invitrogen^TM^), and pGL4.32[luc2P/NF-κB-RE/Hygro] Vector, encoded with the firefly luciferase reporter gene (luc2P) driven by five copies of an NF-*κ*B enhancer element during the first 48 h in culture, and control vector (pGL4.27[luc2P/minP/Hygro]), and in accordance with the manufacturer’s procedure. Equal number of cells was transfected with NF-*κ*B or control luciferase vector. We performed triplicate assays for each treatment condition (bile with and without curcumin and corresponding controls, at pH 4.0 and pH 7.0). At the end of treatments, luminescence was measured using a luminometer (Infinite® M1000 PRO, TECAN) and i-control^TM^ software. We expressed NF-*κ*B activity as ratios of mean values [values for NF-*κ*B reporter (luc2P/NF-kB-RE), against the mean value for control (luc2P)] calculated in treated HHPC for each condition. Finally, we expressed the alterations of NF-*κ*B activity induced by curcumin as ratios of relative NF-*κ*B activity (with/without curcumin) (Data were obtained from three independent experiments).

## **Quantitative real time PCR**

We isolated total RNA (RNeasy mini kit; Qiagen Inc., CA, USA) from HHPC exposed to bile at pH 4.0 and pH 7.0, with or without curcumin, and corresponding controls, to evaluate the transcriptional levels of RELA (p65), c-REL, bcl-2, TNF-α, ΔNp63, EGFR, STAT3, WNT5A, and IL-6, using quantitative real time polymerase chain reaction (qPCR) analysis, as previously described [12,15]. Briefly, we determined RNA quality and concentration by absorption ratios at 260/280 nm (>2.0) and 260 nm, respectively (NanoDrop^TM^ 1000 spectrophotometer; Thermo Fisher Scientific, Waltham, MA). We performed reverse transcription (iScript cDNA synthesis kit; Bio-Rad) and real time qPCR analysis (Bio-Rad real time thermal cycler CFX96TM; Bio-Rad) using specific primers for target genes and reference housekeeping gene, human glyceraldehyde 3-phosphate dehydrogenase (*h*GAPDH) (Table S1), (QuantiTect Primers Assays; Qiagen), and iQ^TM^ SYBR Green Supermix (Bio-Rad). We performed assays in 96-well plates, in triplicate for each sample, and data were analyzed by CFX96^TM^ software. Relative mRNA expression levels were estimated for each target gene relative to reference gene (ΔΔ*C*t). (Data were obtained from three independent experiments).

**PCR array for NF-*κ*B signaling pathway.**

We used a transcriptome of human hypopharyngeal primary cells and a PCR array kit for human NF-*κ*B signaling pathway (RT^2^-Profiler PCR array, PAHS-025z; SABiosciences, Qiagen), following the manufacturer’s instructions. The data were analyzed online by RT^2^-Profiler PCR Array Data Analysis version 3.5 software and differential expression. An estimated 2-fold or more change of gene expression (up and down regulation) was considered significant between the control-treated HHPC (Control group) and HHPC exposed to acidic bile (Group 1) or acidic bile plus curcumin (Group 2). Curcumin-induced up or down-regulation of gene expression was assigned as ratios of relative expression between acidic bile-treated with curcumin group (Group 2), compared to acidic bile-treated group without curcumin (Group 1), normalized to their expression of control treated HHPC (acidic bile+curcumin/control versus acidic bile/control).

## **Cell viability assay**

We performed a cell viability assay, using Cell Titer-Glo® Luminescent Cell Viability Assay (Promega). The cells were seeded at a density 10,000 cells/well for HHPC, respectively, in 24-well plates. The next day the cells underwent repeated exposure for 10 min, two times per day, for 4 days, with experimental (acidic bile at pH 4.0, neutral bile at pH 7.0, with or without curcumin) and control fluid (acid alone at pH 4.0, neutral control at pH 7.0, with or without curcumin). At the end of the treatment, we removed the media and we replaced them with serum free basal media (Human Hypopharyngeal Normal Cell Culture Media Serum Free, for HHPC cells, from Celprogen Inc. CA, USA). Cells were cultured at 37°C in humidified air and 5% CO2 for 12 hours. Subsequently, we used a luminometer to measure the luminescence. All values were normalized to mean value of untreated controls. We determined cell viability by comparing the mean values of cells exposed to NF-*κ*B inhibitor against the mean value for cells not exposed to inhibitor, for each experimental and control group. Supplementary Figure 4S was obtained from three independent experiments. Statistically significant difference of cell viability was determined using paired-test and p value <0.05 (Graph Pad Prism 6.0).


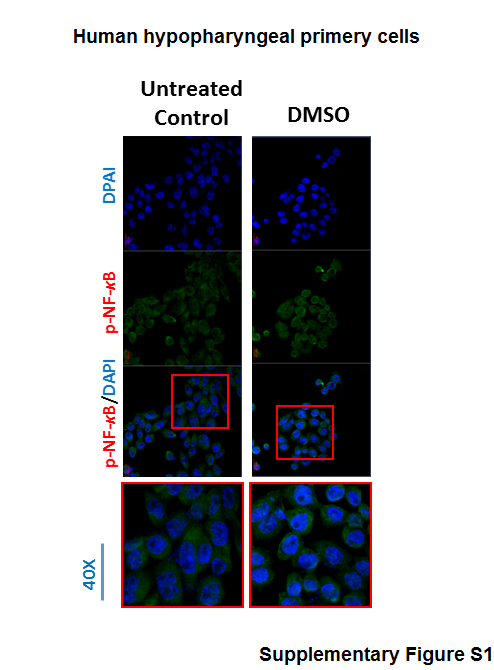


**Supplementary Figure S1: Immunofluorescence staining for phospho-NF-*κ*B (p-p65 Ser536) in DMSO treated and untreated human hypopharyngeal primary cells (HHPC).** DMSO does not affect nuclear translocation of p-p65 (Ser536), in treated HHPC, demonstrating localization of p-p65 similar to untreated HHPC (green: p-p65 Ser536; blue: DAPI for nuclear staining).

**Supplementary Figure S2.** **The inhibitory effect of 50 vs 100 µM curcumin in selected NF-kB related genes of acidic bile-treated human hypopharyngeal primary cells (HHPC).** qPCR analysis reveals that acidic bile-treated HHPC in the presence of 50 µM of curcumin does not generate significant changes in transcriptional levels of RELA(p65) and EGFR, compared to higher concentrations, such as of 100 µM of curcumin. Also, 50 µM of curcumin has a less intense effect than 100 µM of curcumin in reducing the acidic bile-induced mRNA levels of bcl-2, STAT3 and IL-6. (**, *p*<0.005; ***, *p*<0.0005; ****, *p*<0.00005; *t*-test; multiple comparisons by Holm-Sidak; GraphPad Prism 6.0).


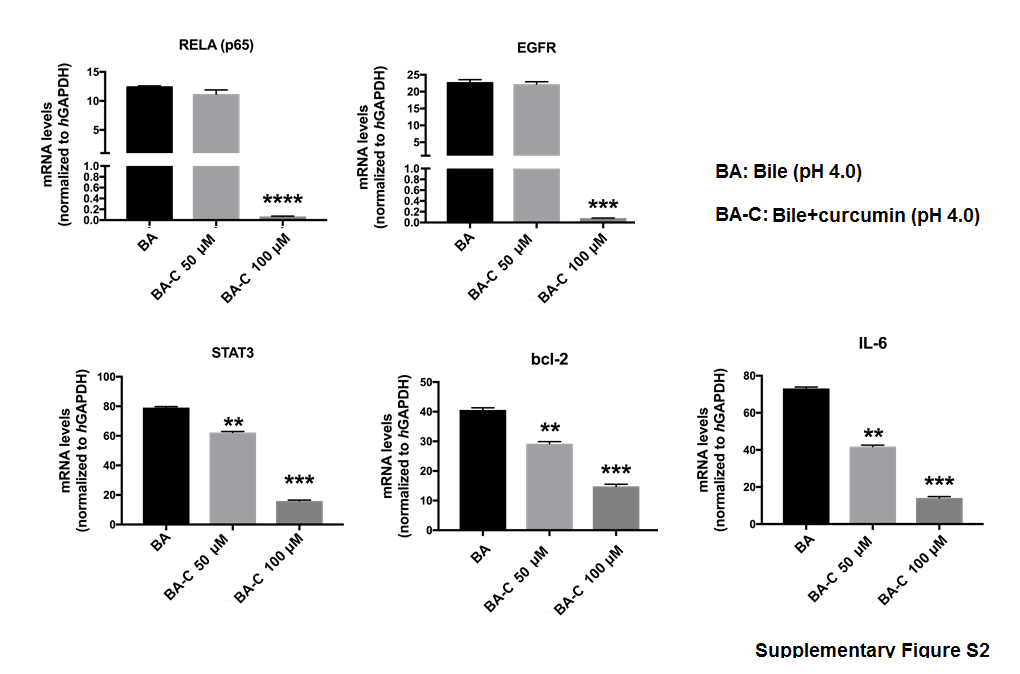


## **Supplementary Figure S3. Correlation by *Pearson* analysis between curcumin-induced transcriptional levels of NF-*κ*B and related genes in HHPC.** *S*trong positive correlation between curcumin-induced **(A)** transcriptionally activated levels of NF-*κ*B (by luciferase assay) and mRNA levels of oncogenic **(a)** EGFR or **(b)** STAT3 (by qPCR), as well as between curcumin-induced **(B)** transcriptional levels of **(a)** RELA(p65) and c-REL, **(b)** RELA(p65) and EGFR, **(c)** RELA(p65) and STAT3, and **(d)** EGFR and STAT3, in HHPC treated groups (by qPCR). (*p* value <0.05).


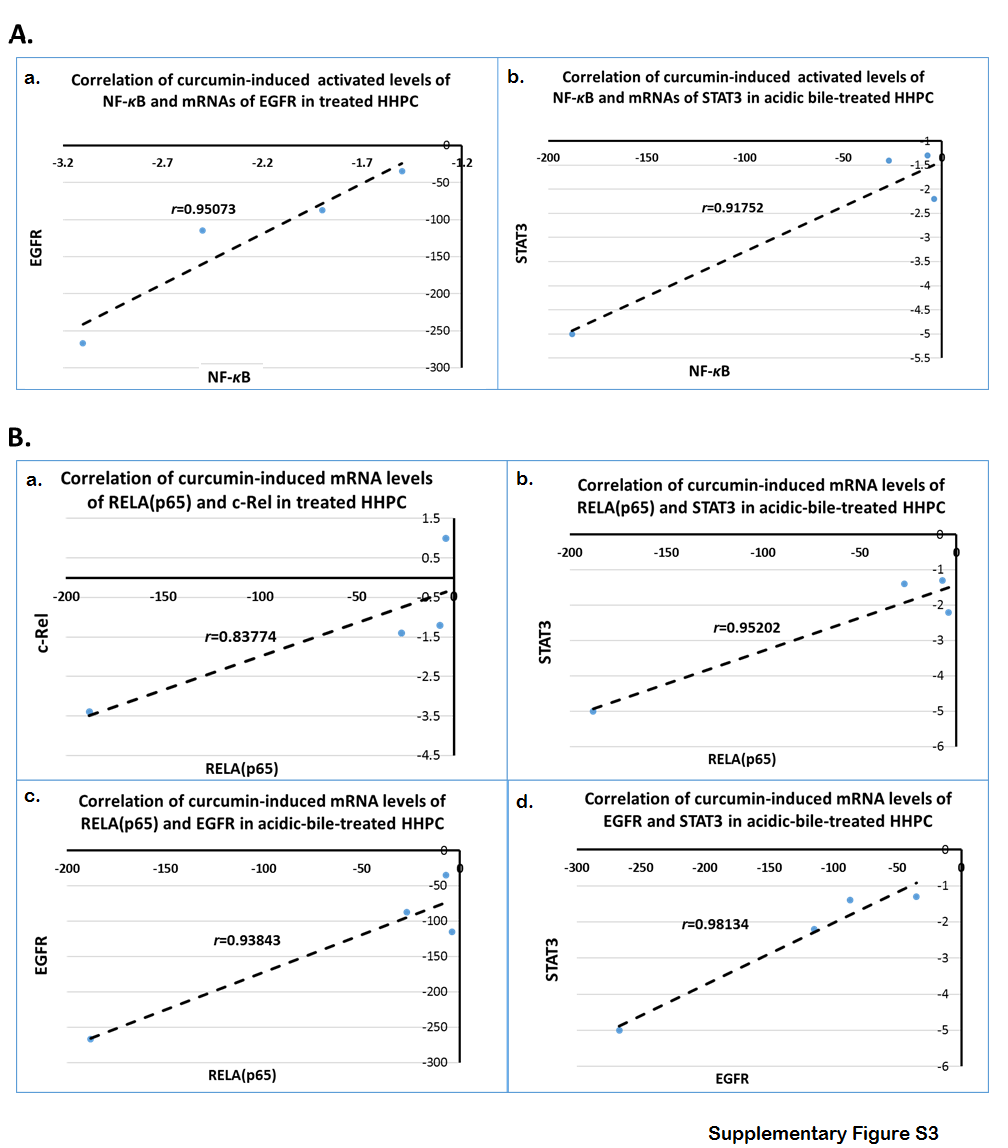


**Supplementary Figure S4. Cell viability assay demonstrates that curcumin (100 μM) reduces the cell viability of HHPC treated with bile at neutral and acidic pH. There nevertheless remains a sufficient percentage of viable cultured cells (A)** Cell viability in HHPC exposed to **(a)** neutral bile and neutral control fluids, **(b)** acidic bile and acid alone, with and without curcumin. **(B)** DMSO had no negative effects on cell viability of treated HHPC, indicated by similar percentages of viable cells compared to controls.


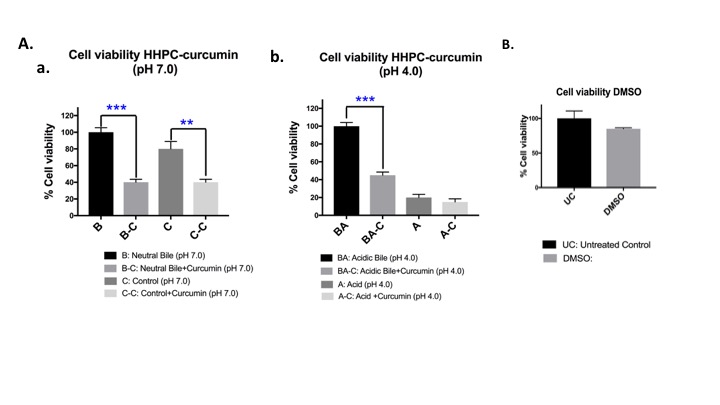


**Supplementary Table S1**: Human genes analyzed by real-time qPCR, in human hypopharyngeal primary cells.

| **Gene** | **Detected transcripts** | **Amplicon length (bp)** |
| --- | --- | --- |
| ***h*GAPDH** | NM_001256799, NM_002046 | 95 |
| **bcl-2** | NM_000633 | 116 |
| **EGFR** | NM_005228  NM_201282-4, | 80 |
| **REL** | NM_002908 | 117 |
| **RELA** | NM_001145138, NM_001243984-5, NM_021975 | 107 |
| **wnt5A** | NM_001256105, NM_003392 | 105 |
| **Tp63** | NM_001114980, NM_003722 | 130 |
| **TNF** | NM_000594 | 98 |
| **STAT3** | NM_003150,  NM_139276 | 95 |
| **IL-6** | NM_000600 XM_005249745 | 107 |

**Supplementary Table S2**: **Down-regulation of NF-*κ*B-related oncogenic pathway in acidic bile with curcumin treated human hypopharyngeal primary cells.**

|  | **HHPC** | | | |
| --- | --- | --- | --- | --- |
|  | **Fold regulation**  **(*mRNA ratios with/without curcumin)** | | | |
| **Target gene** | **Neutral**  **Control (pH 7.0)** | **Neutral Bile**  **(pH 7.0)** | **Acid**  **(pH 4.0)** | **Acidic**  **Bile**  **(pH 4.0)** |
| **bcl-2** | **-2.1** | **-4.7** | **2.0** | **-2.8** |
| **ΔΝp63** | -1.9 | 1.3 | -1.8 | **-3.0** |
| **STAT3** | -1.4 | **-2.2** | -1.3 | **-5.0** |
| **TNF-α** | -1.3 | **-5.8** | **-6.2** | **-9.5** |
| **RELA(p65)** | **-27.0** | **-4.0** | **-7.2** | **-188.0** |
| **wnt5A** | 1.3 | 1.0 | **-8.0** | **-9.7** |
| **EGFR** | **-87.0** | **-115.0** | **-35** | **-267.0** |
| **c-REL** | -1.4 | 1.0 | -1.2 | **-3.4** |
| **IL-6** | **-3.5** | **-11.0** | **-2.4** | **-5.2** |

* Relative mRNA expression ratios (with/without curcumin)

of the analyzed genes were normalized to *h*GAPDH, by qPCR.

**Supplementary Table S3:** Up- or down-regulated genes of NF-*κ*B signaling in HHPC under exposure to acidic bile with and without curcumin.

| **Gene Symbol** | ***BA/Cntl** | ****BA-C/Cntl** | **BA-C/BA** | **Gene Symbol** | **BA/Cntl** | **BA-C/Cntl** | **BA-C/BA** |
| --- | --- | --- | --- | --- | --- | --- | --- |
| AGT | 126.5169 | 73.2674 | -1.7268 | IRF1 | 126.5169 | 73.2674 | -1.7268 |
| AKT1 | 8.5809 | 4.9693 | -1.7268 | JUN | -1.1702 | -2.0206 | -1.7268 |
| ATF1 | 7.5275 | 3.128 | -2.4065 | LTA | 56.587 | 14.3999 | -3.9297 |
| BCL10 | -44.6964 | -77.1811 | -1.7268 | LTBR | 126.5169 | 108.8836 | -1.1619 |
| BCL2A1 | 126.5169 | 73.2674 | -1.7268 | MALT1 | 95.4568 | 48.0823 | -1.9853 |
| BCL2L1 | 2.1147 | 2.1649 | 1.0237 | MAP3K1 | -20.7475 | -35.8265 | -1.7268 |
| BCL3 | 334.0655 | 101.41 | -3.2942 | MYD88 | 24.6842 | 14.2949 | -1.7268 |
| **BIRC2** | 116.3481 | 35.0347 | -3.3209 | NFKB1 | 3.6893 | 2.0393 | -1.8091 |
| BIRC3 | 39.4833 | 22.8652 | -1.7268 | NFKB2 | 2.5318 | -9.3995 | -23.798 |
| CARD11 | 705.1701 | 10.7061 | -65.866 | NFKBIA | -13.3084 | -22.9807 | -1.7268 |
| CASP1 | 241.2826 | 73.2674 | -3.2932 | NFKBIE | 1.2501 | -1.3813 | -1.7268 |
| CASP8 | 126.5169 | 95.3131 | -1.3274 | NOD1 | 429.2685 | 106.3435 | -4.0366 |
| CCL2 | 126.5169 | 73.2674 | -1.7268 | PSIP1 | 126.5169 | 73.2674 | -1.7268 |
| CCL5 | 85.0881 | 20.1608 | -4.2205 | RAF1 | 1.6435 | 1.2007 | -1.3688 |
| CD27 | 17.9154 | 12.6784 | -1.4131 | REL | 126.5169 | 73.2674 | -1.7268 |
| CD40 | 6.5148 | 3.7728 | -1.7268 | RELA | 126.5169 | 73.2674 | -1.7268 |
| CFLAR | 16.7068 | 9.6751 | -1.7268 | RELB | -3.5853 | -6.191 | -1.7268 |
| CHUK | -13.4916 | -54.3948 | -4.0318 | RHOA | 126.5169 | 211.8308 | 1.6743 |
| CSF1 | 42.7338 | 24.7476 | -1.7268 | RIPK1 | 126.5169 | 73.2674 | -1.7268 |
| CSF2 | 547.2337 | 77.047 | -7.1026 | STAT1 | -17.032 | 12.1326 | 206.643 |
| CSF3 | 839.4849 | 262.896 | -3.1932 | TBK1 | 57.0872 | 33.0599 | -1.7268 |
| EGFR | 93.6945 | 54.2595 | -1.7268 | TICAM1 | 100.5568 | 18.9177 | -5.3155 |
| EGR1 | 173.7824 | 83.4909 | -2.0815 | TICAM2 | 126.5169 | 73.2674 | -1.7268 |
| ELK1 | 93.3068 | 54.0351 | -1.7268 | TIMP1 | 16.8127 | 3.9495 | -4.2569 |
| F2R | 2973.749 | 853.7207 | -3.4833 | TLR1 | 126.5169 | 556.1849 | 4.3961 |
| FADD | 126.5169 | 73.2674 | -1.7268 | TLR2 | 142.9153 | 83.0862 | -1.7201 |
| FASLG | 114.9353 | 66.5603 | -1.7268 | TLR3 | 10.8199 | 6.2659 | -1.7268 |
| FOS | -609.193 | -1051.9441 | -1.7268 | TLR4 | 4.4056 | 3.0404 | -1.449 |
| HMOX1 | 48.3911 | 28.0239 | -1.7268 | TLR6 | 107.5202 | 94.8177 | -1.134 |
| ICAM1 | 655.2883 | 171.7173 | -3.8161 | TLR9 | 126.5169 | 73.2674 | -1.7268 |
| IFNA1 | 126.5169 | 73.2674 | -1.7268 | TNF | 126.5169 | 73.2674 | -1.7268 |
| IFNG | 126.5169 | 73.2674 | -1.7268 | TNFAIP3 | 126.5169 | 73.2674 | -1.7268 |
| IKBKB | 126.5169 | 73.2674 | -1.7268 | TNFRSF10A | 126.5169 | 73.2674 | -1.7268 |
| IKBKE | 67.4984 | 59.3361 | -1.1376 | TNFRSF10B | 48.6037 | 63.1242 | 1.2988 |
| IKBKG | -26.6811 | -46.0725 | -1.7268 | TNFRSF1A | 94.9097 | 29.0242 | -3.27 |
| IL10 | 101.1711 | 58.5893 | -1.7268 | TNFSF10 | 267.0436 | 48.9903 | -5.451 |
| IL1A | 28.2689 | 16.3709 | -1.7268 | TNFSF14 | 126.5169 | 73.2674 | -1.7268 |
| IL1B | 126.5169 | 73.2674 | -1.7268 | TRADD | 21.989 | 12.7341 | -1.7268 |
| IL1R1 | 126.5169 | 73.2674 | -1.7268 | TRAF2 | 93.4024 | 54.0904 | -1.7268 |
| CXCL8 | 19.5189 | 11.3036 | -1.7268 | TRAF3 | 226.9448 | 37.455 | -6.0591 |
| IRAK1 | -2.5465 | -1.7027 | 1.4956 | TRAF6 | 126.5169 | 73.2674 | -1.7268 |
| IRAK2 | 9.0996 | 5.2697 | -1.7268 |  |  |  |  |

*BA/Cntl: Relative mRNA expression ratios (acidic bile versus control); **BA-C/Control: Relative mRNA expression ratios (acidic bile plus curcumin versus control); mRNA levels normalized to *h*GAPDH
